# A Transfer Learning-Based VGG-16 Model for COD Detection in UV–Vis Spectroscopy

**DOI:** 10.3390/jimaging11050159

**Published:** 2025-05-17

**Authors:** Jingwei Li, Iqbal Muhammad Tauqeer, Zhiyu Shao, Haidong Yu

**Affiliations:** College of Electrical, Energy and Power Engineering, Yangzhou University, Yangzhou 225100, China; mh23027@stu.yzu.edu.cn (I.M.T.); zhiyushao@yzu.edu.cn (Z.S.); 002740@yzu.edu.cn (H.Y.)

**Keywords:** COD, UV–Vis spectroscopy, GAF, VGG-16, transfer learning

## Abstract

Chemical oxygen demand (COD) serves as a key indicator of organic pollution in water bodies, and its rapid and accurate detection is crucial for environmental protection. Recently, ultraviolet–visible (UV–Vis) spectroscopy has gained popularity for COD detection due to its convenience and the absence of chemical reagents. Meanwhile, deep learning has emerged as an effective approach for automatically extracting spectral features and predicting COD. This paper proposes transforming one-dimensional spectra into two-dimensional spectrum images and employing convolutional neural networks (CNNs) to extract features and model automatically. However, training such deep learning models requires a vast dataset of water samples, alongside the complex task of labeling this data. To address these challenges, we introduce a transfer learning model based on VGG-16 for spectrum images. In this approach, parameters in the initial layers of the model are frozen, while those in the later layers are fine-tuned with the spectrum images. The effectiveness of this method is demonstrated through experiments conducted on our dataset, where the results indicate that it significantly enhances the accuracy of COD prediction compared to traditional methods and other deep learning methods such as partial least squares regression (PLSR), support vector machine (SVM), artificial neural network (ANN), and CNN-based methods.

## 1. Introduction

Water pollution is a critical environmental issue that significantly impacts global sustainable development. It poses severe threats to human health and disrupts the balance of ecosystems, which are essential for maintaining sustainability [1]. Organic pollution is the most common form of contamination in surface waters. High levels of organic pollution reduce dissolved oxygen, endangering aquatic organisms and destabilizing ecosystems [2]. Furthermore, organic contaminants contribute to unpleasant odors, discoloration, foaming, and the presence of toxic small-molecule organic compounds. Long-term exposure to these toxic substances increases the risks of serious health issues, including cancer and teratogenic [3]. Reliable scientific indicators are crucial for the effective assessment and monitoring of organic pollution. Chemical oxygen demand (COD) serves as a key metric for measuring organic pollution, as it quantifies the oxygen consumed during the oxidation of organic matter under controlled conditions [4,5]. COD is widely used to evaluate water pollution levels and is a critical parameter in water quality assessments [6]. Higher COD values reflect a higher concentration of reducing substances and indicate more severe pollution. Therefore, rapid and accurate COD detection is essential for routine water quality assessments, sewage treatment, and tracking pollution sources.

UV–Vis spectroscopy is the most commonly used method for assessing water quality due to its ability to provide comprehensive spectral data [7]. The technique enables the collection of full-spectrum information, which is essential for developing advanced spectral analysis algorithms. This rich dataset enhances the accuracy and applicability of spectral analysis in water quality research. One of the key advantages of UV–Vis spectroscopy is its rapid analysis capability, allowing for quick results. Additionally, it is cost-effective, making it a practical solution for routine water quality assessments. The method is particularly well suited for fast evaluations and continuous environmental monitoring, making it effective for real-time tracking of water quality changes. This combination of speed, low cost, and high accuracy makes UV–Vis spectroscopy an invaluable tool in water quality studies [8].

After acquiring spectral data, establishing an accurate relationship between spectral features and COD values is essential for accurate water quality assessment. Machine learning techniques are widely employed to model spectral data and address nonlinear variations in absorption spectra. Several studies have compared different machine learning models to identify the most effective approach [9,10]. Cao et al. [11] conducted a comprehensive study comparing four different modeling approaches for COD prediction. These models included multiple linear regression (MLR), partial least squares regression (PLSR), least squares support vector machine (LS-SVM), and backpropagation artificial neural network (BP-ANN). Among these approaches, BP-ANN demonstrated the best performance, achieving the highest predictive accuracy. To enhance the efficiency of BP-ANN, feature selection was performed using the successive projections algorithm (SPA). This method identified 11 key wavelengths, reducing redundant information while preserving essential spectral features. The optimized BP-ANN model exhibited strong predictive capabilities. The coefficient of determination (R^2^) reached 0.90. The root mean squared error (RMSE) reached 10.96 mg/L. Additionally, the model achieved a ratio of performance to deviation (RPD) of 5.06. These results confirm that BP-ANN, in combination with SPA-based feature selection, is an effective approach for COD estimation using UV–Vis spectroscopy. Li et al. [12] proposed a practical method for COD detection based on UV–Vis spectroscopy. To ensure broad applicability, the COD estimation model was globally calibrated using effluents from rural sewage treatment devices. This approach aimed to improve model generalization and adaptability to real-world wastewater samples. To evaluate the effectiveness of different modeling techniques, three machine learning methods were compared: PLSR, SVM, and BP-ANN. Each method was assessed based on its predictive accuracy and robustness in COD estimation. Among these approaches, PLSR demonstrated the best performance, achieving a high level of accuracy while maintaining computational efficiency. Lyu et al. [13] proposed a comprehensive framework that integrates both desktop and in situ UV–Vis spectrometers to develop reliable spectral fitting models. This framework enables accurate estimation of multiple water quality parameters, ensuring consistency between laboratory and field measurements. The study aimed to improve the accuracy of in situ spectroscopic measurements by leveraging data from a high-precision desktop spectrometer. The developed models focused on predicting key water quality indicators, including nitrate-nitrogen (NO_3_-N), total nitrogen (TN), COD, total phosphorus (TP), and suspended solids (SS). Each parameter was estimated using different spectroscopic modeling approaches to ensure optimal predictive performance. In the case of COD estimation, principal component regression (PCR) utilizing all available wavelengths yielded the most accurate results. Xu et al. [14] introduced a novel reflective detection system designed for non-contact measurement of UV–Vis absorption spectra to estimate COD in water. Unlike conventional transmissive detection systems, this approach eliminates direct sample contact, reducing contamination risks and enhancing system durability. To assess the effectiveness of the reflective detection system, the study compared its modeling results with those obtained from a transmissive detection system. The performance of both systems was evaluated using multiple regression analysis algorithms, including PLSR, SVM, and random forest (RF). These algorithms were applied to develop predictive models for COD estimation. Additionally, the study explored the impact of various spectral preprocessing techniques and different feature selection bands on the accuracy of the models. These preprocessing steps played a crucial role in enhancing the robustness and reliability of the system. The reflective detection system was thoroughly validated using standard water samples, demonstrating high accuracy with an R^2^ of 0.98892. The system also achieved a low RMSE of 2.8677 mg/L, indicating accurate COD predictions. Zhou et al. [15] proposed a novel method for predicting COD and turbidity using ultraviolet-visible-near infrared (UV–Vis–NIR) absorption spectrum. This method leveraged a BP-ANN to establish a relationship between spectral absorbance and water quality parameters. The BP-ANN model was trained using absorbance values measured within the 240–1040 nm wavelength range as input data. The corresponding COD and turbidity values from the training set served as the output. The training process involved datasets of varying sizes to assess how the number of training samples influenced model performance. To evaluate the generalization ability of the trained model, the test set’s original absorbance values without any compensation were used to make predictions. The results demonstrated that an increase in the training sample size significantly enhanced the model’s ability to fit the data. The optimal training sample size was found to be 2400 samples, ensuring reliable predictions. The proposed BP-ANN-based approach achieved high predictive accuracy. The correlation coefficient exceeded 0.9998, indicating a strong linear relationship between predicted and actual values. The RMSE for COD prediction was as low as 0.19 mg/L, while for turbidity prediction, the RMSE was 0.34 NTU. However, traditional machine learning models often suffer from poor generalization, limiting their effectiveness in COD estimation [16]. Deep learning has emerged as a promising alternative due to its ability to self-learn and extract features autonomously, reducing the reliance on manual feature engineering. Its layered processing structure enables it to capture complex spectral mapping relationships, making it well suited for COD prediction and addressing challenges associated with spectral analysis [17].

Deep learning has increasingly replaced traditional methods in water quality analysis due to its ability to extract features and improve predictive accuracy automatically. Among various deep learning approaches, convolutional neural networks (CNNs) have demonstrated significant potential in COD prediction [18]. Jia et al. [19] proposed a novel approach for COD prediction by integrating a CNN with ultraviolet–visible (UV–Vis) spectroscopy. This method leverages the feature extraction capabilities of CNN to improve prediction accuracy. To enhance data quality, the Savitzky–Golay smoothing filter was employed to mitigate noise interference in spectral signals. The CNN model follows a structured feature extraction process. First, the convolution layer captures essential spectral features through localized filtering operations. Next, the pooling layer reduces the feature dimensions, preserving critical information while minimizing computational complexity. Finally, the fully connected layer integrates extracted features to generate the final COD prediction. The proposed CNN model demonstrated superior predictive performance. It achieved high accuracy in COD estimation, with predictions closely aligning with the regression curve. Comparative evaluations against other models confirmed its advantages. CNN obtained the smallest root mean square error of prediction (RMSEP) and mean absolute error (MAE), indicating lower prediction deviations. Additionally, it achieved the highest R^2^, reflecting a stronger correlation between predicted and actual COD values. Ye et al. [20] proposed a COD prediction model that integrates UV–Vis spectrometry with a CNN. This approach effectively addresses the challenge of predicting COD in water with complex pollutant compositions. Unlike traditional models, which rely on the selection of specific wavelengths, this method utilizes the full absorbance spectrum. It avoids potential information loss and enhances prediction accuracy by preserving all spectral information. The model architecture is designed based on a shallow CNN. Instead of traditional pooling layers, it employs convolutional layers with varying step lengths. This design reduces computational complexity while improving the ability to capture spectral feature peaks. The CNN model also demonstrates strong feature extraction capabilities, minimizing dependence on extensive preprocessing techniques. As a result, it enhances the effective use of spectral information and improves overall predictive performance. Comparative analysis confirms the superiority of this approach. It consistently outperforms traditional COD prediction models, including principal component analysis (PCA), PLSR, and BP-ANN. Xia et al. [21] introduced a rapid COD retrieval method for absorption-fluorescence spectra to address errors associated with COD retrieval in the UV–Vis absorption spectrum method, particularly in fluorescent organic matter solutions. The algorithm development involved the integration of a 1D-CNN with a two-dimensional Gabor transform, resulting in a novel absorption-fluorescence spectrum fusion neural network algorithm. This fusion approach aimed to enhance the accuracy of COD retrieval from complex water samples. In tests using an amino acid aqueous solution, the absorption-fluorescence method achieved a relative root mean square error of prediction (RRMSEP) of 0.32%, which represented an 84% reduction compared to the single UV–Vis spectrum method. Additionally, the accuracy of COD retrieval was 98%, showing a 15.3% improvement over the UV–Vis spectrum approach. Guan et al. [22] introduced a significant improvement to traditional COD detection models by integrating deep learning techniques with a spectrum preprocessing algorithm. This innovative approach aims to enhance the performance of COD prediction by addressing key challenges, such as noise sensitivity, which is often a limitation in conventional models. To mitigate noise interference, the model incorporated an improved noise filter based on discrete wavelet transforms. This method effectively reduces the impact of noise, ensuring more reliable and accurate detection of COD levels in complex samples. In addition to noise handling, the study proposed a novel COD detection network designed to overcome the issue of poor generalization often seen in traditional models. The model achieved better adaptability to various datasets by enhancing the network architecture, improving its overall robustness. Experimental results demonstrated the effectiveness of the proposed method. The model showed excellent performance, not only exhibiting strong tolerance to noise but also achieving high accuracy in COD prediction, thus confirming the practical advantages of integrating deep learning with advanced spectrum preprocessing techniques. In these studies, one-dimensional convolutional neural networks were used to train samples, and the sample set size used was only a few hundred to a few thousand, with each sample reaching several thousand dimensions. Such a small sample set is difficult to fully train deep CNNs, which can easily lead to overfitting of the models. These challenges hinder their adoption in this field. Training deep CNNs requires large labeled datasets, which are often scarce in water quality research. Insufficient data can lead to overfitting, reducing the generalization ability of the model [23]. Furthermore, labeling water quality data is a labor-intensive and costly process, so most collected data remains unlabeled. Additionally, training very deep CNNs demands substantial computational resources. The high processing power and time required to train these models from scratch make their practical implementation difficult. It is essential to address these limitations for further advanced deep learning-based COD prediction based on UV–Vis spectroscopy. Therefore, studying CNN models suitable for small sample sizes and high dimensions is one of the important ways to improve COD detection accuracy.

Transfer learning has emerged as a powerful strategy to overcome challenges in tasks with limited data by leveraging pre-trained models. This approach involves using knowledge from one pre-trained model and applying it to a new task [24,25]. The primary benefit of transfer learning is that pre-trained CNNs can extract universal low-level features from large datasets, which are useful for many tasks, even when task-specific datasets are small. Fine-tuning a pre-trained model for a specific task can also significantly reduce both training time and computational requirements. Several pre-trained CNN models, such as VGGNet, GoogLeNet, and ResNet, have demonstrated success across various fields [26,27]. For instance, Bansal et al. [28] applied the VGG-16 model to develop an AI-based solution for geographical landmark recognition, successfully transferring knowledge from ImageNet and creating a highly generalized model. In the field of fault classification. Prasshanth et al. [29] compared 15 pre-trained models, including DenseNet-201 and GoogLeNet, to classify faults in monoblock centrifugal pumps. They achieved the highest classification accuracy by fine-tuning hyperparameters, showcasing the effectiveness of pre-trained models in industrial applications.

The objective of this paper is to enhance both the speed and accuracy of COD detection in water based on UV–Vis spectroscopy. Although existing CNN models have achieved good results in image recognition, they cannot be directly used for spectral datasets. The main reason is that spectral data is a one-dimensional vector, while existing CNNs are mainly used for two-dimensional images. Secondly, it is difficult to establish sufficient spectral datasets to train such deep models. To overcome these challenges, this paper proposes a preprocessing method to convert one-dimensional UV–Vis spectra into two-dimensional images and performs feature extraction and modeling based on the VGG-16 model and transfer learning. The VGG-16 model is initially pre-trained on the ImageNet dataset, which allows the model to learn general features. In order to save the pre-training time of VGG-16, the pre-trained model parameters with a top-5 accuracy of 90.382% in PyTorch were directly loaded. Following this, the CNN is fine-tuned by a small, preprocessed UV–Vis spectroscopy dataset, shifting the model’s focus from image recognition to spectrum image recognition. This paper presents an innovative method that converts one-dimensional spectra into two-dimensional images, marking a significant advancement in the field. By transforming the spectral data into a visual format, the potential to apply advanced image recognition techniques is unlocked, which has shown great success in various domains. These transformed images are then processed using state-of-the-art image recognition algorithms, enabling a more efficient and accurate analysis of the data. Additionally, the challenge of limited training data for deep learning models is effectively tackled through the application of transfer learning. This technique leverages pre-trained models, thus enhancing the performance of the model even with a smaller dataset. Together, these contributions offer a novel approach to spectral data analysis and provide a robust framework for addressing challenges in deep learning applications. The method is validated through a series of experiments, and the results demonstrate satisfactory COD prediction performance. This approach offers efficiency benefits by minimizing the time and resources typically required for collecting and labeling large spectroscopy datasets, making it a promising solution for water quality monitoring.

This paper is organized as follows: Section 2 presents the materials and methods utilized throughout the research. Section 3 describes the experimental procedures conducted and provides a comprehensive analysis of the obtained results. Section 4 offers a critical discussion of the experimental findings. Section 5 concludes the paper by summarizing the major findings and contributions of the study.

## 2. Materials and Methods

In this section, we present a detailed description of the materials and methods utilized in our paper. Firstly, we describe the source of the samples, the process of collecting the UV–Vis spectra, and the measurement of the COD standard values. Secondly, we propose the spectral preprocessing and modeling methods used for analysis. Finally, we introduce the evaluation indicators utilized to assess the quality and performance of the developed model.

### 2.1. Dataset Acquisition

#### 2.1.1. Water Sample Collection

The water samples in this study were collected from the Grand Canal in Yangzhou, a historically significant waterway with over 2000 years of history. The canal plays a crucial role in supporting local ecosystems, particularly fish and bird habitats, and contributes to the region’s water resources and environmental health. However, the water quality of the canal faces significant challenges due to the discharge of sewage from urban development and industrial activities. As a result, effective water quality detection and real-time monitoring are essential to ensure the well-being of local residents and maintain ecological balance. Water samples were collected continuously from various regions of the canal over one year, from January 2024 to December 2024. Sampling was conducted four times a day, excluding holidays, yielding a total of 1000 samples. To evaluate the performance of the proposed COD prediction model, the collected data were split into a training/calibration set and a testing/prediction set at a ratio of 4:1. To maintain a similar distribution between the two sets, an equidistant sampling method was applied. The dataset was first sorted in ascending order based on COD standard values. Every fifth sample was selected for the testing set, while the remaining samples were assigned to the training set. Consequently, the sequence numbers of the testing set samples were 3, 8, 13, …, 998. This approach resulted in a training/calibration set of 800 samples and a testing/prediction set of 200 samples. These division results are commonly used in machine learning applications.

#### 2.1.2. Spectral Acquisition

**Figure 1:**
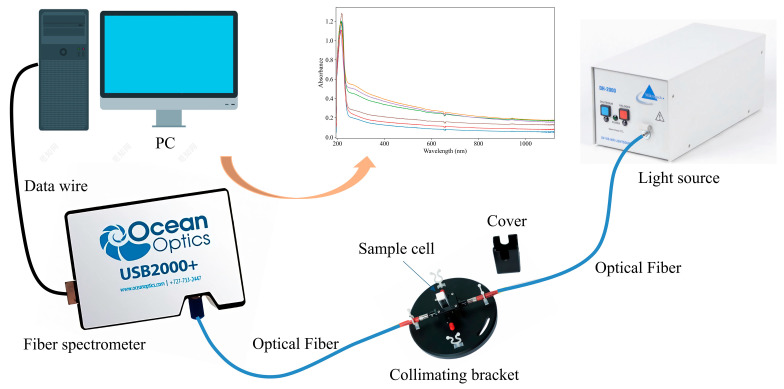
UV–Vis spectrum collecting system.

**Figure 2:**
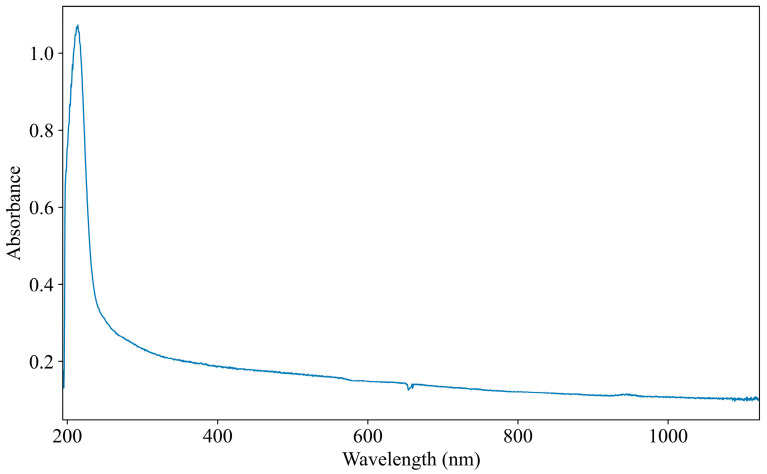
One spectrum of the 1000 samples.

The UV–Vis spectroscopy acquisition system consists of several key components, including a light source, sample cell, spectrometer, and a computer for data processing, as shown in Figure 1. The light source employed in the system is a DH-2000 deuterium-halogen light source, which emits light across a broad range of 190 to 2500 nm (Ocean Optics, Orlando, FL, USA). The sample cell, with an optical path length of 10 mm, allows for efficient transmission of light through the water sample. The USB2000+ spectrometer, which measures wavelengths from 165 to 1200 nm with a resolution of 0.48 nm, is employed to capture the spectral data (Ocean Optics, Orlando, FL, USA). Data acquisition and management are handled by OceanView software 2.0.8, which stores full wavelength spectra or data from specific wavelength ranges. The collected data is processed and modeled by a computer, with appropriate software applied for further analysis. One of the UV–Vis spectra obtained from the water samples is shown in Figure 2, providing a visual representation of the data for COD analysis.

Figure 2 illustrates a UV–Vis absorbance spectrum obtained from the sample. The horizontal axis represents the wavelength range, spanning from 193.61 nm to 1121.69 nm. This broad spectral range enables the capture of key absorption features associated with COD detection. The spectrum is recorded with a high resolution of approximately 0.48 nm, resulting in a total of 2048 discrete wavelength points. This fine resolution ensures the accurate representation of spectral characteristics. The vertical axis denotes absorbance values, which range from 0.10 to 1.07, reflecting variations in light absorption at different wavelengths. A prominent absorption peak is observed near 250 nm, corresponding to the primary absorption feature of COD. Additionally, a distinct groove appears at 660 nm. This anomaly is caused by spectrometer noise, which is inherent to the measurement system.

#### 2.1.3. COD Standard Value Measurement

The COD of the water samples was determined by the rapid digestion spectrophotometry method, which is known for its efficiency and accuracy. The measurement was conducted by a DRB200 digester (HACH, Loveland, CO, USA) and a DR3900 visible spectrophotometer (HACH, Loveland, CO, USA). First, the required chemical reagents were carefully mixed with the water samples to ensure proper chemical reactions. The samples were then placed in a preheated DRB200 COD digester set at 165 °C for 20 min to allow for complete digestion. After the digestion process, the tubes were removed and allowed to cool to room temperature (25 ± 1 °C) to stabilize the samples. Finally, a color reagent was added to each tube, and the COD values were measured by the DR3900 spectrophotometer, providing accurate results for water quality assessment. The COD statistical results of the sample set are shown in Table 1.

### 2.2. VGG-16 Architecture

**Figure 3:**
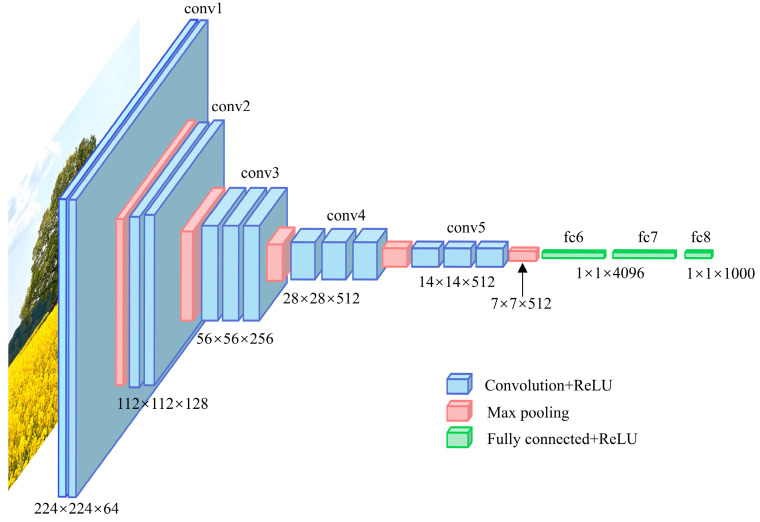
The standard architecture of VGG-16 model.

VGG-16 is a prominent convolutional neural network developed by the Visual Geometry Group (VGG) at the University of Oxford in 2014 [30]. It has achieved impressive results in numerous image-processing tasks and remains one of the most effective models today. The architecture of VGG-16 consists of 21 layers, which include 13 convolutional layers, 5 pooling layers, and 3 fully connected layers, as shown in Figure 3. The name “VGG-16” originates from the fact that only 16 layers, comprising the convolutional and fully connected layers, contain weights. The model has 64 filters in the first convolutional layer, 128 filters in the second, 256 filters in the third, and 512 filters in both the fourth and fifth layers. One of the key design points of VGG-16 is its use of small 3 × 3 convolutional filters, which, combined with its deep architecture, enable it to effectively capture intricate features. Initially designed for image classification, VGG-16 gained significant recognition for its high accuracy in the ImageNet competition. Its straightforward design and effectiveness have made it a popular choice for a wide range of computer vision tasks. Additionally, VGG-16 serves as a foundation for many transfer learning applications, demonstrating its versatility in various domains [31,32].

### 2.3. The Proposed Method

#### 2.3.1. Spectrum Pre-Processing Based GAF

Traditional UV–Vis spectral feature extraction operates in a one-dimensional space (vector), which limits its ability to capture the overall morphological information embedded in the spectrum. This restriction makes it challenging to fully represent the complexity of the data. To overcome this limitation, the paper proposes a method that transforms one-dimensional spectral data into a two-dimensional matrix, or image. This conversion enables the data to better capture the global information and underlying relationships present in the spectrum. To extract meaningful features from these transformed spectrum images, deep learning CNNs are employed. CNNs offer a modern alternative to traditional machine learning techniques, significantly enhancing the ability to extract features from UV–Vis spectral data. This approach improves both feature extraction and modeling, allowing for a more comprehensive understanding of the data and better prediction performance.

There are two primary approaches for converting one-dimensional sequence data into two-dimensional images: decomposition and transformation [33]. Decomposition methods, such as Fourier transform and wavelet transform, break down a spectrum into multiple feature vectors of varying frequencies. These vectors are then combined to form a matrix, which can be visualized as a grayscale image [34]. However, the differing dimensions in the transformed image can complicate subsequent feature extraction, limiting the effectiveness of these methods. On the other hand, transformation techniques, such as recurrence plot (RP) [35], Markov transition fields (MTF) [36], and Gramian angular field (GAF) [37], offer an alternative approach to transforming one-dimensional vectors into two-dimensional matrices. These transformation methods have been successfully applied in various fields, including vibration signal analysis, electrocardiograms, and the analysis of physiological signals. This paper focuses on the GAF method as a representative example of the transformation process. The GAF algorithm works by transforming each value in a sequence into an angle in polar coordinates. It then generates an image by calculating the sine and cosine of these angles. The conversion of one-dimensional UV–Vis spectra into two-dimensional images based on the GAF method involves three key steps, which are central to the process of transforming the data for better analysis.

Step 1: The normalization of the one-dimensional spectral sequence X is an important step in the process, where the values are scaled to the interval [−1, 1]. This normalization is performed by the method shown in Equation (1). After normalization, the spectral sequence is mapped to the polar coordinate system. In this mapping process, each normalized value ${\tilde{x}}_{i}$ is associated with a polar angle $\psi_{i}$, and the corresponding wavelength $\lambda_{i}$ is mapped to the radius r. The scaled sequence is now represented in the polar coordinate system, as shown in Equation (2).

(1) $${\tilde{x}}_{i}=\frac{\left(x_{i}-\max\left(X\right)\right)+\left(x_{i}+\min\left(X\right)\right)}{\max\left(X\right)-\min\left(X\right)}$$

(2) $$\left\{\begin{array}{c} \psi_{i}=\arccos\left({\tilde{x}}_{i}\right),{\tilde{x}}_{i}\in \left[-1,1\right]\\ \;r=\frac{\lambda_{i}}{N}\;\;\;\;\;\;\; \end{array}\right.$$

where X is the one-dimensional sequence. $x_{i}$ is a single value in the sequence. ${\tilde{x}}_{i}$ is the normalized value of $x_{i}$. $\psi_{i}$ is the polar angle after the inverse cosine transform. r is the polar diameter. $\lambda_{i}$ is wavelength. N is the constant factor for adjusting the span of the polar coordinate system.

Step 2: The difference and sum operations, based on sine and cosine functions, are applied to transform the spectral data into two-dimensional matrices known as the Gramian angular difference field (GADF) and the Gramian angular sum field (GASF). GADF is obtained by calculating the sine value of the difference. GASF is obtained by calculating the cosine value of the sum. The mathematical representations for these operations are provided in Equation (3) for the GADF and in Equation (4) for the GASF.

(3) $$GADF=\left[\begin{array}{c} \sin\left(\psi_{1}-\psi_{1}\right)\;\sin\left(\psi_{1}-\psi_{2}\right)\ldots \sin\left(\psi_{1}-\psi_{n}\right)\\ \sin\left(\psi_{2}-\psi_{1}\right)\;\sin\left(\psi_{2}-\psi_{2}\right)\ldots \sin\left(\psi_{2}-\psi_{n}\right)\\ \;\;\;\;\;\vdots \;\;\;\;\;\;\;\;\;\;\;\;\vdots \;\;\;\;\;\ddots \;\;\;\;\;\;\;\vdots \;\;\;\\ \sin\left(\psi_{n}-\psi_{1}\right)\;\sin\left(\psi_{n}-\psi_{2}\right)\ldots \sin\left(\psi_{n}-\psi_{n}\right) \end{array}\right]\;={\sqrt{I-{\tilde{X}}^{2}}}^{T}\tilde{X}-{\tilde{X}}^{T}\sqrt{I-{\tilde{X}}^{2}}$$

(4) $$GASF=\left[\begin{array}{c} \cos\left(\psi_{1}+\psi_{1}\right)\;\cos\left(\psi_{1}+\psi_{2}\right)\ldots \cos\left(\psi_{1}+\psi_{n}\right)\\ \cos\left(\psi_{2}+\psi_{1}\right)\;\cos\left(\psi_{2}+\psi_{2}\right)\ldots \cos\left(\psi_{2}+\psi_{n}\right)\\ \;\;\;\;\;\vdots \;\;\;\;\;\;\;\;\;\;\;\;\vdots \;\;\;\;\;\ddots \;\;\;\;\;\;\;\vdots \;\;\;\\ \cos\left(\psi_{n}+\psi_{1}\right)\;\cos\left(\psi_{n}+\psi_{2}\right)\ldots \cos\left(\psi_{n}+\psi_{n}\right) \end{array}\right]\;={\tilde{X}}^{T}\tilde{X}-{\sqrt{I-{\tilde{X}}^{2}}}^{T}\sqrt{I-{\tilde{X}}^{2}}$$

where I is the unit vector.

Step 3: Each value in the obtained matrix is scaled to a range between 0 and 255 to standardize the data. This scaling process ensures that the values are within an appropriate range for image representation. The scaling is performed by Equation (5). After scaling, the two-dimensional matrix is transformed into a GAF, which is represented as a grayscale image.

(5) $$L\left(j,k\right)=int(127.5(GAF\left(j,k\right)+1))$$

where L (j, k) is the pixel value at point (j, k) in the image. int (∙) is the rounding function. GAF (j, k) is the element value corresponding to the jth row and kth column of the GADF or GASF matrix.

**Figure 4:**
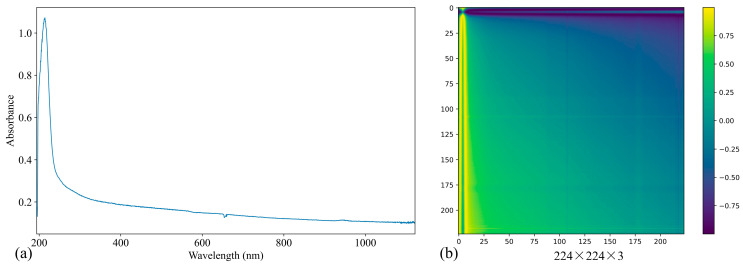
One-dimensional UV–Vis spectrum transform into two-dimensional image based on GASF. (**a**) Original spectrum. (**b**) Spectrum image.

An example of transforming a one-dimensional UV–Vis spectrum into a two-dimensional image is shown in Figure 4b, with the original spectrum displayed in Figure 4a. To meet the input requirements of the CNN, specifically VGG-16, the grayscale images need to be transformed into color images. This is achieved by applying a color map, such as Viridis, to the grayscale images. The color map enhances the visual representation of the data, making it suitable for processing by CNN. Additionally, the color images are resized to 224 × 224 × 3 to ensure compatibility with the input dimensions required by VGG-16. This resizing process standardizes the images, allowing them to be fed into the model for feature extraction and subsequent analysis.

#### 2.3.2. Transfer Learning

Spectral images were created based on preprocessing, thus transforming the spectral modeling task into an image modeling task. This conversion allows the application of advanced image processing techniques to spectroscopy data. However, one challenge that arises is the risk of overfitting, particularly due to the limited amount of data available. To mitigate this risk, pre-trained CNNs are used. Instead of training a new CNN model from scratch, deep learning leverages pre-trained models, such as those trained on large datasets like ImageNet, which helps address the scarcity of training data. Transfer learning is a key approach in this context. It involves adapting the weights of a well-trained model to a new model that solves a related problem, reducing the need for large datasets [38]. In this study, transfer learning was applied by using a pre-trained VGG-16 CNN model. Certain layers of the model were frozen, minimizing the need for extensive fine-tuning and allowing the model to perform effectively despite the small dataset.

**Figure 5:**
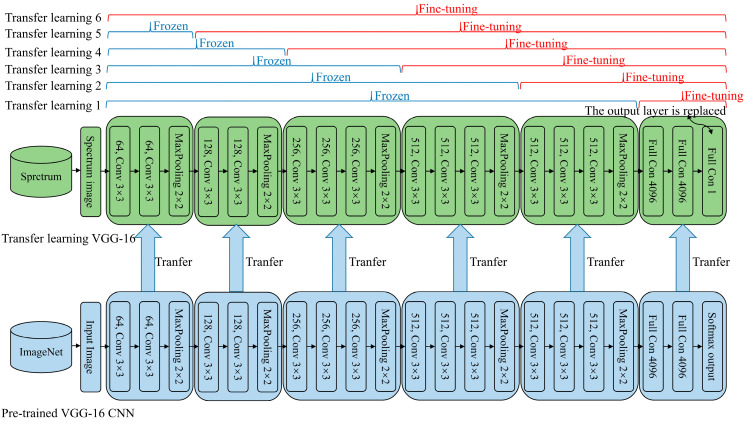
The proposed transfer learning network based on VGG-16.

The weights of the transfer learning model play a crucial role in ensuring good performance, even with a small dataset, as long as the dataset used to train the pre-trained model is sufficiently large. In this approach, only the later layers of the new model are trained. This training strategy is effective in reducing the need for extensive data while maintaining model performance. Transfer learning offers several key benefits. It significantly reduces training time and the amount of labeled data required for the new task. Low-level features from the early layers of the model are generally applicable across a variety of tasks, whereas high-level features from the later layers are more task-specific [39,40]. The front layers of the model serve as universal feature extractors, which can be applied to many different tasks, enhancing the model’s versatility. The proposed method employs the VGG-16 model with transfer learning for processing spectrum images. This model consists of a pre-trained VGG-16 CNN for extracting universal features. A gradual thawing strategy is employed in this approach, where the later layers are fine-tuned first, followed by the gradual adjustment and fine-tuning of the front layers. This strategy optimizes the model’s parameters, ensuring that the best transfer learning model is achieved. Figure 5 illustrates the details of the transfer learning-based network based on VGG-16 researched in this study.

#### 2.3.3. Fine-Tuning

The proposed transfer model closely resembles the pre-trained VGG-16 CNN, with one key modification: the output layer. The original fully connected layer, which contains 1000 neurons, is replaced by a new layer consisting of a single neuron for regression tasks. This modification ensures that the model is suitable for continuous value prediction, such as in the case of COD detection from spectrum images. Structures and weights from the pre-trained CNN model are transferred to the new model, improving its performance and allowing for more efficient learning. This approach enables fine-tuning of the VGG-16 model for the spectrum image task without the need for complete retraining, significantly reducing computation time. To further refine the model, the mean squared error (MSE) loss function is employed during fine-tuning. The MSE loss function, as defined in Equation (6), ensures that the model’s predictions closely match the actual values by minimizing the square of the differences between predicted and true values.

(6) $$MSE=\frac{1}{n}\sum_{i=1}^{n}{\left(y_{i}^{t}-y_{i}^{p}\right)}^{2}$$

where n is the number of samples. $y_{i}^{t}$ is the measured value based on the standard method. $y_{i}^{p}$ is the predicted value based on the proposed method.

The experimental setup is conducted with PyTorch GPU 2.5.1, taking advantage of dual GeForce RTX 4090 GPUs (Taiwan Semiconductor Manufacturing Company, Taiwan, China) to accelerate the training process. The environment also includes CUDA 11.8 for parallel processing, a 2.9 GHz Intel^®^ Xeon^®^ Silver 4310 CPU (Intel’s semiconductor fabrication plants, Chandler, USA), and 32 GB of RAM for general computations. The operating system employed is Windows 10, with Python 3.9.6 as the programming language, ensuring compatibility and stability in running the model training and evaluation processes.

### 2.4. COD Prediction Process of the Proposed Method

**Figure 6:**
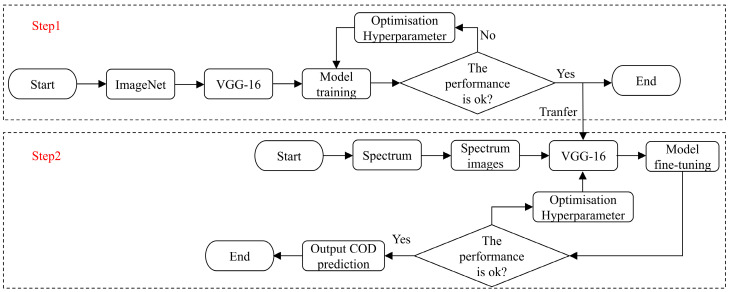
COD prediction process of the proposed method.

The training process consists of two main steps: pre-training the VGG-16 CNN and fine-tuning the transfer learning model for COD prediction. Initially, the VGG-16 CNN is pre-trained on the ImageNet dataset to extract general low-level features that can be applied across various tasks. To reduce the overall training time, a pre-trained deep learning model was utilized in this study. The VGG-16 model used in this work was initialized with pre-trained weights loaded from PyTorch. The pre-trained VGG-16 model achieves a top-5 recognition accuracy of 90.382% on ImageNet, indicating its high capacity for feature extraction and generalization. By directly loading these pre-trained weights into the current task, the need for training the model from scratch is eliminated. Once the pre-training is complete, the model is initialized for transfer learning. This initialization helps the model adapt to the specific task of COD prediction. The obtained spectrum images are then split into training and testing datasets. The training dataset is employed for fine-tuning the transfer learning model, while the testing dataset is reserved for evaluating its performance. Since the fine-tuning dataset differs from ImageNet, additional layers of the transfer CNN need to be fine-tuned. Various fine-tuning schemes are tested, and the most effective one is selected based on experimental results. A layer freezing strategy is applied, where the front layers of the transfer CNN are frozen, and only the later layers are fine-tuned to minimize the loss function. Once fine-tuning is complete, the optimal network is applied to UV–Vis spectrum images for COD prediction. The entire training procedure is illustrated in Figure 6.

### 2.5. Performance Indices

Different modeling methods for COD prediction offer various strengths and weaknesses, making it essential to evaluate and compare them effectively. To facilitate this comparison, standardized quantitative indices are necessary. Several evaluation indices are commonly used to assess model performance, including the coefficient of determination (R^2^) and the root mean square error for calibration (RMSEC) and prediction (RMSEP). The interpretation of these evaluation indices is straightforward. Higher R^2^ values indicate a stronger correlation between the predicted and actual COD values, while lower RMSEC and RMSEP values suggest better model accuracy and less error. Conversely, lower R^2^ values and higher RMSEC/RMSEP values mean poorer model performance. The equations for calculating these performance metrics are provided in Equations (7)–(9).

(7) $$R^{2}=1-\frac{\sum_{i=1}^{n}{\left({\hat{y}}_{i}-y_{i}\right)}^{2}}{\sum_{i=1}^{n}{\left(y_{i}-\bar{y}\right)}^{2}}$$

(8) $$RMSEC=\sqrt{\frac{1}{n^{c}-1}\sum_{i=1}^{n^{c}}{\left({\hat{y}}_{i}^{c}-y_{i}^{c}\right)}^{2}}$$

(9) $$RMSEP=\sqrt{\frac{1}{n^{p}}\sum_{i=1}^{n^{p}}{\left({\hat{y}}_{i}^{p}-y_{i}^{p}\right)}^{2}}$$

where $y_{i}$ is the measured value according to the standard method. $\bar{y}$ is the average of $y_{i}$; ${\hat{y}}_{i}$ is the predicted value from the model. n is the number of samples. $y_{i}^{c}$ is the measured value from the calibration set. ${\hat{y}}_{i}^{c}$ is the predicted value from the calibration model. $n^{c}$ is the number of samples in the calibration set. $y_{i}^{p}$ is the measured value from the prediction set. ${\hat{y}}_{i}^{p}$ is the predicted value from the prediction model. $n^{p}$ is the number of samples in the prediction set.

## 3. Experiments and Results Analysis

In this section, we provide a detailed description of the experiments and results analysis in this paper. Firstly, we studied the optimal model and hyperparameter selection. Then, we described the fine-tuning process of the method proposed in this paper. Finally, the performance of our method in COD prediction was described and compared with other methods.

### 3.1. Selection of Model

The pre-trained model with a top-5 accuracy of 90.382% was initialized and utilized as the foundational framework for all experiments. Various transfer learning schemes were applied to the model. These schemes involve freezing and fine-tuning different network layers of the model as different transfer learning models. The experimental evaluation was conducted on the 1000 sample datasets we collected. To ensure reliable and generalizable performance assessment, 10-fold cross-validation was employed. The original dataset is randomly shuffled and evenly divided into 10 subsets (called folds) based on a specific random seed, each with an equal sample size. Use the first fold as the validation set, and merge the remaining 9 folds into the training set (fine-tuning set). Loop until all folds serve as validation sets. Each validation set must record the same evaluation index (RMSE). Calculate the mean and standard deviation of 10 results. The initial hyperparameter settings are as follows: Adam is selected as the optimizer, with a learning rate of 0.001 to ensure smooth convergence. The training process is conducted with a batch size of 24 and spans 200 epochs without early stops to facilitate comprehensive learning. Based on the aggregated results of the cross-validation, the most effective transfer learning scheme was selected. The performance of each transfer learning model was carefully analyzed, and the results are summarized in Table 2, providing a clear comparison of the models’ effectiveness in COD prediction.

As the number of fine-tuning layers increases from transfer learning model 1 to 4, the model’s performance improves. Specifically, the mean of RMSEs decreases. This indicates that the model improves at predicting COD values as more layers are trained. The improvement occurs because the later layers of the model focus on learning task-specific features, which allows for better adaptation to the new task. However, as the number of training layers increases from transfer learning model 4 to 6, the performance begins to decline. The mean of RMSEs increases, suggesting that the model’s ability to predict COD values deteriorates. This decline is likely caused by overfitting, which occurs when the entire model is trained on small datasets. In contrast, training only the later layers helps reduce model complexity and improves generalization to new tasks. The standard deviations of these transfer learning models are all very small and not significantly different, so the stability of these models is good. The optimal model 4 achieved excellent performance, with the mean of RMSEs being 4.3801 and the standard deviation of RMSEs being 0.1656.

### 3.2. Selection of Hyperparameters

Adjusting hyperparameters is a critical step in optimizing deep learning models after confirming that transfer learning mode 4 is the optimal model. Proper hyperparameters directly affect model performance, training stability, and convergence speed. In this study, two key hyperparameters are analyzed: learning rate and batch size. These parameters play a fundamental role in determining the effectiveness and efficiency of model training. The learning rate is an essential hyperparameter in optimization algorithms. It controls the step size for updating model parameters during backpropagation. A learning rate that is too high can cause unstable training, leading to divergence. Conversely, a learning rate that is too low may result in slow convergence or getting stuck in local minima. Selecting an appropriate learning rate is crucial for achieving optimal performance. The batch size defines the number of samples used to compute gradients in a single training iteration. A small batch size allows for more frequent updates, potentially improving model generalization but increasing training noise. A large batch size stabilizes updates and can speed up training on powerful hardware, but it may require careful tuning to avoid convergence issues. Balancing batch size is essential for efficient model training. Different combinations of these two hyperparameters are tested to evaluate their influence on model convergence and performance. The experimental results, including the cross-validation performance for various learning rate and batch size settings, are presented in Table 3.

Learning rate and batch size have a significant influence on COD prediction performance in deep learning models. A well-chosen learning rate ensures stable and efficient training, while an appropriate batch size balances computational efficiency and model generalization. The results of Table 3 reveal that the optimal combination is a learning rate of 0.0001 and a batch size of 32. The mean and standard deviation of RMSEs are minimized under this combination. This configuration achieves the best balance between training/fine-tuning stability and prediction accuracy.

### 3.3. Fine-Tuning Procedure

**Figure 7:**
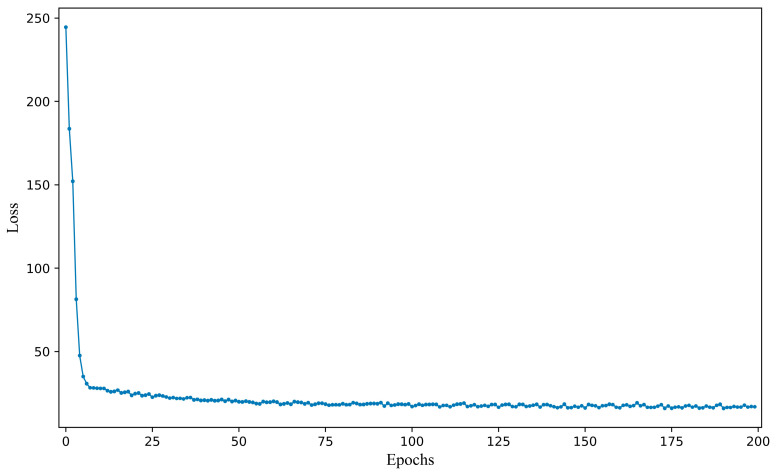
Changes in loss value during the fine-tuning process of transfer learning model 5.

The transfer learning model 4 was fine-tuned after determining the optimal hyperparameters. The Adam optimization algorithm was employed to optimize the network parameters during the fine-tuning process. To ensure that the best-performing model was employed during the testing stage, the loss was saved at the end of each epoch, as shown in Figure 7. The loss value exhibited a significant decrease in the first six epochs and began to stabilize around the 100th epoch, indicating that the model was gradually converging. After 100 epochs, the loss value remained largely unchanged, indicating that the model was approaching convergence. The relatively quick convergence of the model can be attributed to the small sample size and the simple mapping relationship between the UV–Vis spectra and the COD values, which facilitated the learning process. Despite the model having converged, the final loss value only decreased to around 15, rather than reaching 0, which can be attributed to factors such as noise and turbidity in the spectra, which introduced some interference during fine-tuning.

### 3.4. Visualization of Feature Importance

**Figure 8:**
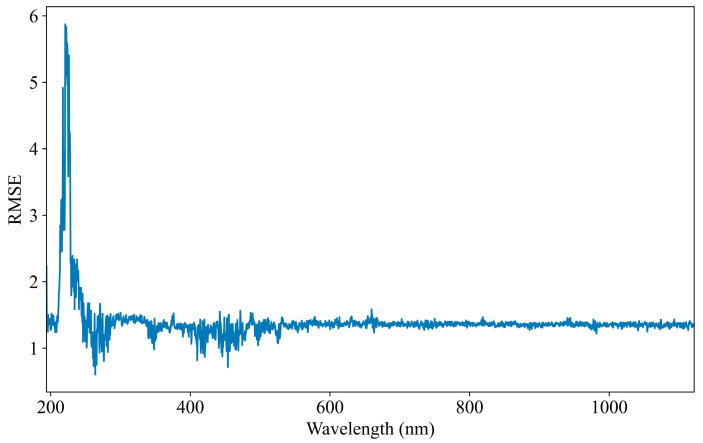
UV–Vis spectral importance curve based on permutation feature importance.

The evaluation of feature importance is essential for understanding the contribution of each input variable to COD prediction performance. This study assesses the significance of individual features within the fine-tuned model’s prediction set. Identifying key features enhances model interpretability and helps optimize input selection for improved accuracy. To quantify feature importance, the permutation feature importance (PFI) method is employed. PFI is a model-agnostic technique that measures the influence of each feature by randomly shuffling its values and observing the corresponding change in model performance. A greater increase in prediction error indicates a more influential feature, while minimal change suggests a less significant contribution. RMSE is used as the primary evaluation index. Higher RMSE values after feature permutation indicate a stronger dependence of the model on the corresponding feature. To facilitate analysis, the feature importance results are visualized in Figure 8. This graphical representation highlights the relative significance of each feature, offering valuable insights into their role in COD prediction.

The feature importance analysis reveals a strong correlation between the spectral feature importance curve (Figure 8) and the absorption spectrum (Figure 2). It is worth noting that the most critical spectral region in Figure 8 that has the greatest impact on COD prediction is aligned with the main absorption peak around 250 nm in Figure 2. This indicates that the model is effectively identifying and prioritizing the relevant features that are crucial for accurate COD prediction. The model’s ability to automatically extract features related to COD further confirms its capacity to learn and adapt to important spectral patterns. By focusing on the main absorption peak, the model captures essential information that directly correlates with COD levels in the water samples. However, it is also observed that the model occasionally learns irrelevant features, such as the 660 nm peak, which is likely a result of spectrometer noise rather than any meaningful COD-related information. This extraction of irrelevant features can negatively influence the model’s accuracy. The inclusion of such noise introduces unwanted variability into the prediction process, reducing the model’s accuracy in detecting COD concentrations. Moreover, non-COD-related factors, such as turbidity and organic salts, may introduce further interference in the UV–Vis spectroscopy data. These external factors lead to inaccuracies in the COD detection process, highlighting the need to carefully consider potential influencing factors when training and deploying models for real-world applications.

### 3.5. Model Performance Analysis

**Figure 9:**
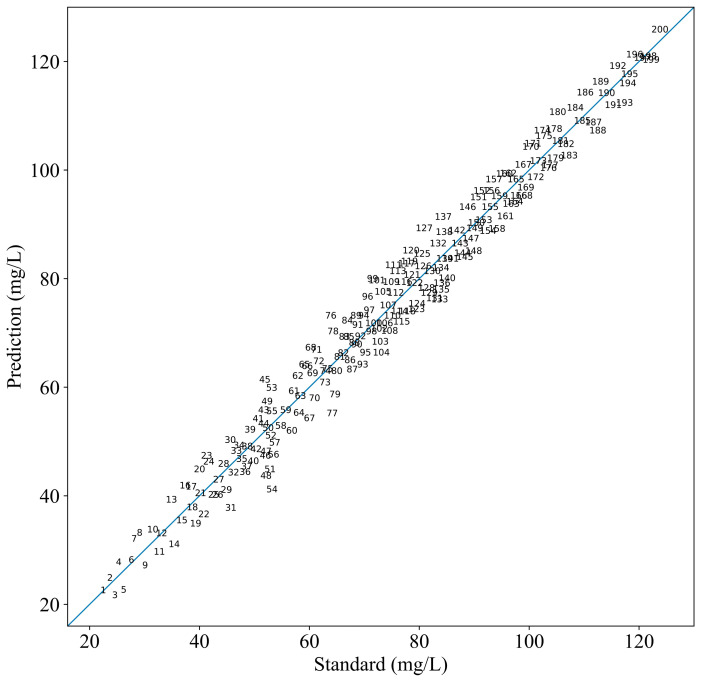
Comparison curve between the prediction and standard COD values.

After the fine-tuning stage, the model that achieved the lowest loss value was saved as the final COD prediction model, ensuring that the most optimized version of the model was employed for further testing. The saved model was then tested by the 200 testing/prediction set. The predicted COD values from the model were compared with the standard COD values to assess the model’s performance. A summary of the model’s performance, including various evaluation indices, is provided in Table 4. To quantify the accuracy of the model’s predictions, a linear analysis was conducted by comparing the predicted COD values with the standard COD values. The results of this linear fitting analysis are shown in Figure 9.

The linearity between the predicted and standard values was shown in Figure 9, with higher accuracy indicated by the scatter points being closer to the fitted line. The strong agreement between the predicted and standard values suggests that the model possesses good robustness and adaptability, making it highly reliable for accurate COD prediction in water samples. Specifically, the proposed method demonstrated a clear linear relationship between the predicted and standard COD values, with the scatter points closely following the straight line that has a slope of 1. Importantly, no significant error growth was observed as the COD concentration increased, indicating that the model performs consistently across a wide range of COD concentrations, particularly between 20 and 120 mg/L. This stability in error distribution further highlights the generalization ability of the model. The experiments confirm that the method effectively employs spectral information from across the entire UV–visible spectrum, ensuring comprehensive feature extraction for accurate COD predictions.

### 3.6. Comparison with Other Methods

The effectiveness of the proposed method was systematically evaluated by comparing its prediction and fitting accuracy against both traditional and deep learning methods for COD prediction. To ensure a comprehensive assessment, three widely recognized traditional methods were selected for comparison. These included PLSR [13], SVM [12], and ANN [41]. Each of these models has been extensively used in COD prediction and spectral data analysis, making them suitable benchmarks for performance evaluation. In addition to traditional methods, three CNN-based deep learning models [19,20,22] were included in the comparative analysis. These CNN networks were chosen based on their frequent application in spectral predictive modeling tasks. Deep learning models have demonstrated superior feature extraction capabilities, making them promising candidates for COD detection. The cross-validation performances are presented in Table 5.

The performance comparison of different COD prediction methods is presented in Table 5. These results highlight the differences between the proposed method, traditional models, and various deep learning approaches. Among traditional methods, the ANN demonstrates the best performance, achieving a mean RMSE of 8.7612 and a standard deviation of 0.3015. However, despite its relatively strong performance, ANN still struggles to fully capture the nonlinear relationship between spectral data and COD values. Other traditional methods, such as PLS and SVM, perform worse than ANN. They exhibit poorer fitting results and higher prediction errors, as reflected in their larger mean and standard deviation of RMSEs.

In contrast, deep learning methods show improved predictive capabilities. The CNN model from previous studies [22] emerges as the most effective deep learning approach among the three deep learning methods. It achieves a mean RMSE of 5.6150, demonstrating better accuracy compared to traditional methods. Simultaneously obtained a smaller standard deviation than the other two CNN models. However, the other two deep learning methods fail to outperform this CNN model, highlighting inefficiencies in capturing the complex spectra-COD relationship.

The proposed method surpasses all previously mentioned methods, including PLS, SVM, ANN, and CNN-based approaches. It attains a lower mean of RMSEs of 4.2318, and the standard deviation of RMSEs is 0.1528, indicating higher accuracy and stability in COD estimation. Its ability to extract meaningful spectral features makes it a highly reliable method for COD prediction, outperforming both traditional and deep learning-based alternatives.

## 4. Discussion

Training a deep CNN such as VGG-16 from scratch presents significant computational challenges. It requires substantial processing power and a large dataset to achieve optimal performance. However, utilizing a pre-trained model can effectively mitigate these issues. Models trained on large datasets, such as ImageNet, offer a significant advantage by reducing training time. Instead of learning from raw data, only the final layers require fine-tuning for a specific task. This approach is particularly beneficial for UV–Vis spectral analysis, where datasets are often limited in size. Training a deep model directly on small spectral datasets increases the risk of overfitting, reducing model reliability. Transfer learning provides a robust solution by leveraging features learned from extensive datasets, thereby improving generalization even when spectral data is scarce. One-dimensional spectral data is transformed into two-dimensional images to apply CNNs effectively to spectral analysis. This conversion allows the network to exploit spatial feature extraction capabilities, enhancing spectral recognition. The pre-trained VGG-16 model is particularly effective in this context. Its well-structured convolutional architecture improves feature extraction, enabling more accurate and efficient identification of spectra compared to training from scratch.

The proposed method demonstrates high effectiveness in COD detection using UV–Vis spectroscopy. Its performance is thoroughly evaluated through comparative analysis, as presented in Table 5. The model achieves outstanding prediction accuracy, with the mean of RMSEs being 4.2318 and the standard deviation of RMSEs being 0.1528. These low error values indicate high accuracy in estimating COD concentrations. These results confirm its ability to effectively capture complex relationships between spectral features and COD values. Furthermore, comparisons with existing models in the literature highlight the superior performance of the proposed method. Deep learning-based methods significantly outperform traditional techniques such as PLS, SVM, and ANN. One key advantage of deep learning is its capability to model complex, nonlinear relationships in spectral data. Unlike traditional approaches, which often struggle with intricate spectral variations, deep learning algorithms extract meaningful features more efficiently. These findings strongly reinforce the effectiveness of deep learning-based models in COD prediction, demonstrating their potential for accurate and reliable water quality monitoring.

The proposed method significantly improves the accuracy of COD detection using UV–Vis spectroscopy. It effectively captures spectral features and establishes a reliable prediction framework. However, despite its high accuracy, the method faces challenges in generalization. When applied to different surface waters or industrial wastewater, its prediction performance declines. This limitation arises because the model is specifically fine-tuned on a particular dataset, restricting its ability to adapt to new water samples. The fine-tuning process has optimized the model for specific conditions, making it difficult to generalize relationships for unseen data. To enhance its applicability across diverse water sources, further fine-tuning is necessary. This requires collecting additional water samples from various environments and systematically adjusting the model. Incorporating data from different water bodies would strengthen its adaptability and improve prediction accuracy in broader applications. Although the model demonstrates strong performance within its current dataset, modifications are essential to enhance its robustness across varying water types. Future research should focus on expanding the dataset and refining the model to ensure more reliable COD detection in diverse environmental conditions.

## 5. Conclusions

The paper presents a novel method for predicting water COD based on a deep transfer CNN network in conjunction with UV–Vis spectroscopy. A GAF-based data preprocessing technique is proposed, which transforms one-dimensional spectra into two-dimensional gray-scale images. Each spectrum image is then transformed into a 224 × 224 × 3 color image, making it compatible with the VGG-16 architecture. A transfer learning approach is introduced to address overfitting issues that arise from limited training data. This approach leverages a pre-trained VGG-16 CNN, initially trained on the ImageNet dataset, and transfers its weights and structures to fine-tune the CNN by the transformed spectrum images. The transfer of pre-trained model weights not only improves prediction accuracy but also accelerates the training process. The proposed method is applied to UV–Vis spectrum modeling and COD prediction, with experiments conducted on a dataset of 1000 samples. Experimental results demonstrate that the proposed method outperforms traditional and deep learning methods, such as PLSR, SVM, ANN, and CNNs, in terms of accuracy. The robust feature extraction capabilities of CNNs significantly reduce the reliance on traditional COD modeling techniques. As a result, the method achieves improved fitting and higher prediction accuracy, offering an effective solution for rapid and accurate COD detection in water.

## Figures and Tables

**Table 1:** COD statistical results of the sample set.

Sample Set	Samples	Mean (mg/L)	Minimum (mg/L)	Maximum (mg/L)	Standard Deviation (mg/L)
Training set	800	67.86	23.1	128.4	27.59
Testing set	200	67.42	24.3	126.2	27.12
All	1000	67.57	23.1	128.4	27.41

**Table 2:** Evaluation of the transfer learning models’ prediction effect.

Method.	μ	σ
Transfer learning mode 1	8.3619	0.1661
Transfer learning mode 2	7.5197	0.1689
Transfer learning mode 3	5.6497	0.1685
Transfer learning mode 4	4.3801	0.1656
Transfer learning mode 5	5.0113	0.1755
Transfer learning mode 6	6.2498	0.1717

μ: The mean of RMSEs; σ: the standard deviation of RMSEs.

**Table 3:** Cross-validation performance of the model with the combination of learning rate and batch size.

Learning Rate	Batch Size									
	8		16		32		64		128	
	μ	σ	μ	σ	μ	σ	μ	σ	μ	σ
0.00005	4.6412	0.1924	4.5679	0.1547	4.3935	0.1539	4.2818	0.1620	4.7392	0.1608
0.0001	5.3089	0.1743	4.4464	0.1672	4.2318	0.1528	4.5026	0.1542	5.1408	0.1535
0.0005	5.4709	0.1937	5.9191	0.1643	4.7652	0.1542	4.4704	0.1535	5.7828	0.1596
0.001	6.1552	0.1734	4.3801	0.1656	5.1627	0.1643	5.0266	0.1673	5.0808	0.1658
0.003	6.5127	0.2038	5.3841	0.1734	5.1104	0.1618	4.8098	0.1595	4.8264	0.1729
0.005	5.7071	0.1967	6.1594	0.2172	6.1593	0.1706	5.3534	0.1632	4.7859	0.1794
0.01	6.3373	0.2126	6.5447	0.2237	5.3286	0.1943	5.1603	0.1741	5.1867	0.1672

μ: The mean of RMSEs; σ: the standard deviation of RMSEs.

**Table 4:** The performance of the proposed method.

Method	Calibration Set		Prediction Set	
	R^2^	RMSEC	R^2^	RMSEP
Proposed method	0.9783	3.8834	0.9751	4.1662

**Table 5:** Cross-validation performance of the proposed method and other methods.

Method	μ	σ
PLSR	11.2885	0.4318
SVM	9.7082	0.3726
ANN	8.7612	0.3015
CNN [19]	6.3985	0.2164
CNN [20]	5.8186	0.2656
CNN [22]	5.6150	0.1752
Proposed method	4.2318	0.1528

μ: The mean of RMSEs; σ: the standard deviation of RMSEs.

## Data Availability

The data that support the findings of this study are available from the corresponding author.
